# Supplementary material for: Application of PEG-Covered Non-Biodegradable Polyelectrolyte Microcapsules in the Crustacean Circulatory System on the Example of the Amphipod Eulimnogammarus verrucosus
Source: Polymers (Basel). 2019 Jul 27;11(8):1246. doi: 10.3390/polym11081246 (PMC6723028; doi:10.3390/polym11081246)
Supplement: Supplementary file 1 [file polymers-11-01246-s001.pdf]

Supplementary Material

to the article

Application of PEG-covered non-biodegradable polyelectrolyte microcapsules in the crustacean circulatory system on the example of the amphipod *Eulimnogammarus verrucosus*

by

Ekaterina Shchapova, Anna Nazarova, Anton Gurkov, Ekaterina Borvinskaya, Yaroslav Rzhechitskiy, Ivan Dmitriev, Igor Meglinski, Maxim Timofeyev

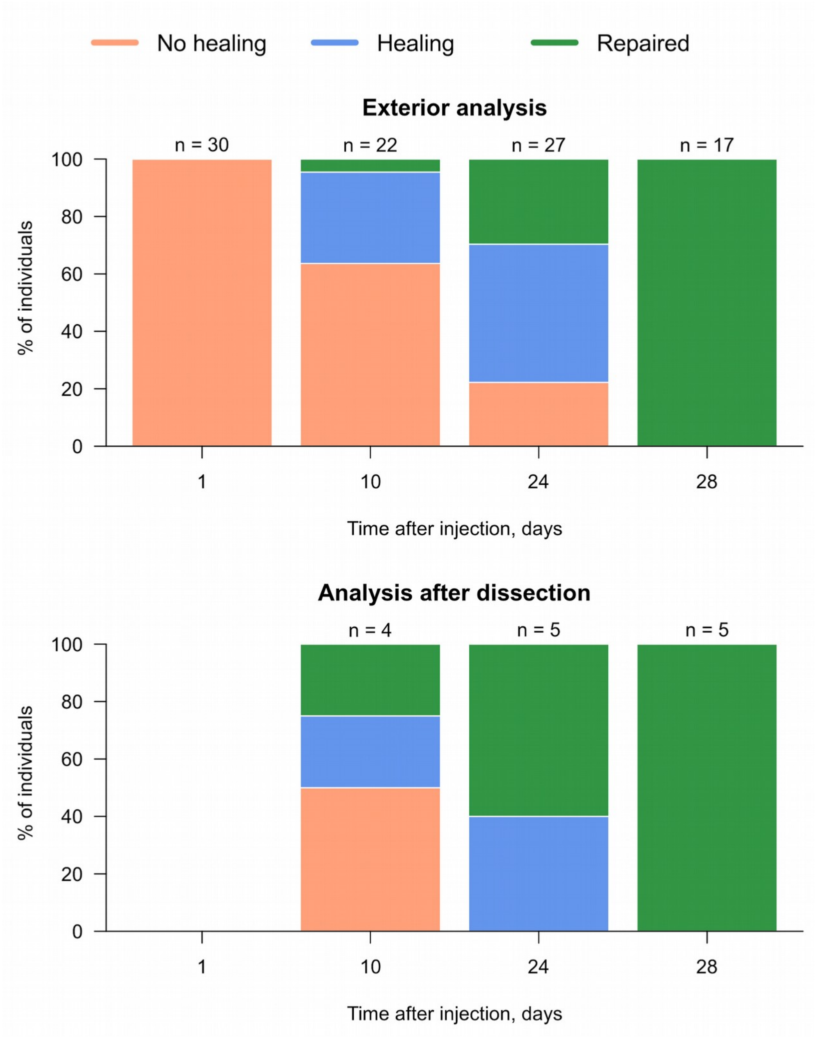

Figure S1. Checkup of *E. verrucosus* exoskeleton integrity after injection between the sixth and seventh segments of pereon (area of the central hemolymph vessel). Analyses included exterior inspection (upper panel) and examination after dissection (lower panel).

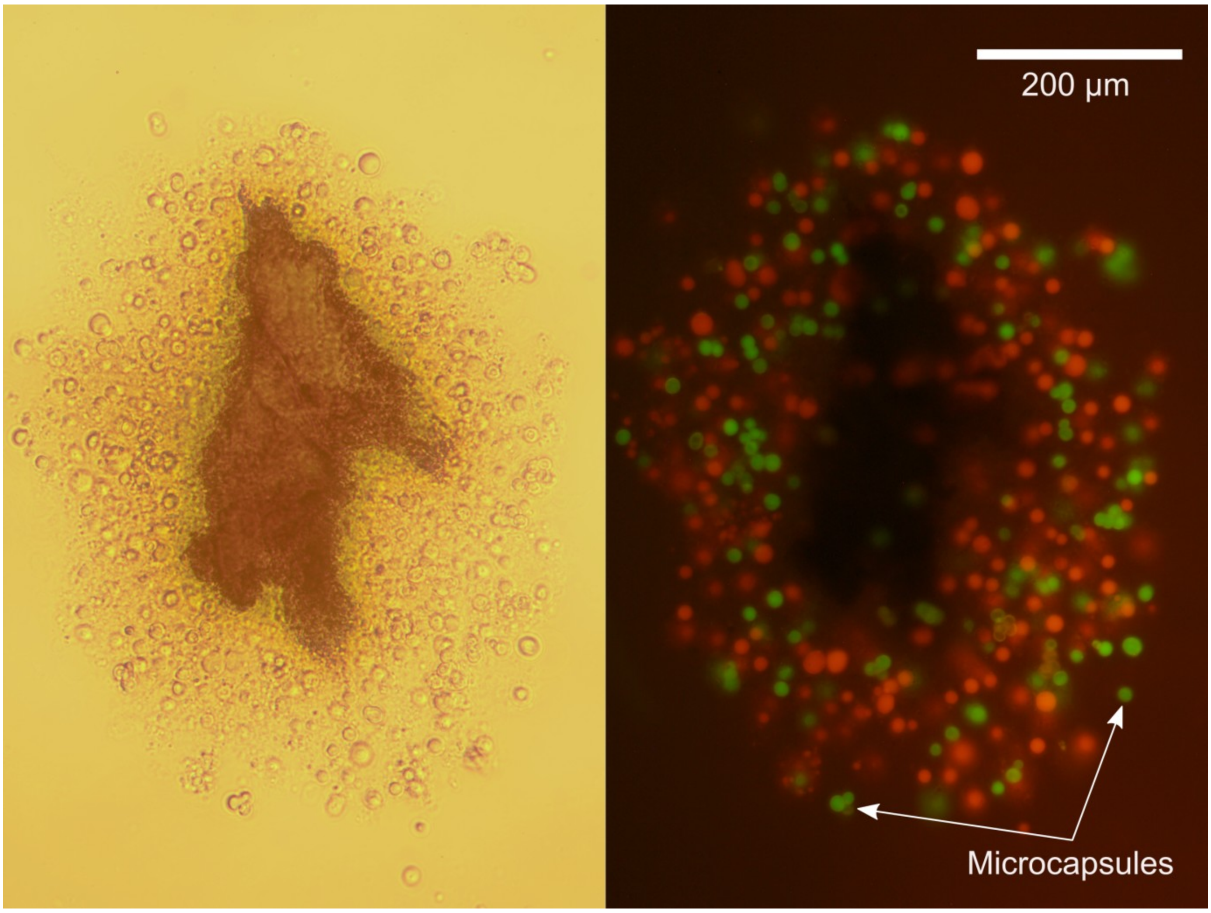

Figure S2. Melanization (dark area in the center) inside an aggregate of hemocytes with microcapsules two days post introduction of microcapsules into the culture media. Visualization in brightfield channel (left panel) and green fluorescent channel with the orange $RuPhen_3$ staining (right panel). Microcapsules contained green FITC-albumin, but partially obtained the yellow coloration due to contact with $RuPhen_3$.

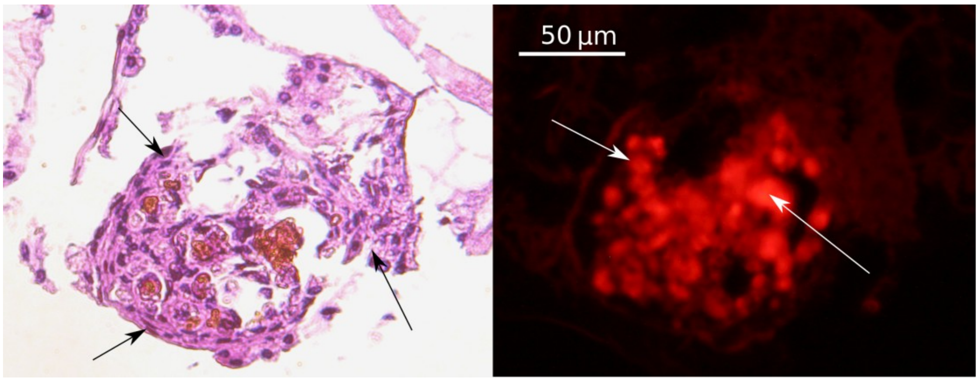

Figure S3. Example of hemocyte accumulation around an aggregate of microcapsules one week post injection into the central hemolymph vessel of *E. verrucosus*. Histological section of amphipod tissue is visualized in the brightfield channel after hematoxylin and eosin stain (left panel) and in the red fluorescent channel (right panel). Microcapsules contained SNARF-1-dextran. Black arrows indicate hemocytes; white arrows indicate microcapsules.
